# Molds with Advanced Materials for Carbon Fiber Manufacturing with 3D Printing Technology

**DOI:** 10.3390/polym13213700

**Published:** 2021-10-27

**Authors:** Patrich Ferretti, Gian Maria Santi, Christian Leon-Cardenas, Marco Freddi, Giampiero Donnici, Leonardo Frizziero, Alfredo Liverani

**Affiliations:** Department of Industrial Engineering, Alma Mater Studiorum University of Bologna, I-40136 Bologna, Italy; gianmaria.santi2@unibo.it (G.M.S.); marco.freddi@studio.unibo.it (M.F.); giampiero.donnici@unibo.it (G.D.); leonardo.frizziero@unibo.it (L.F.); alfredo.liverani@unibo.it (A.L.)

**Keywords:** FDM, chemical smoothing, vapor smoothing, PVB, carbon fiber mold

## Abstract

Fused Deposition Modeling (FDM) 3D printing is the most widespread technology in additive manufacturing worldwide that thanks to its low costs, finished component applications, and the production process of other parts. The need for lighter and higher-performance components has led to an increased usage of polymeric matrix composites in many fields ranging from automotive to aerospace. The molds used to manufacture these components are made with different technologies, depending on the number of pieces to be made. Usually, they are fiberglass molds with a thin layer of gelcoat to lower the surface roughness and obtain a smooth final surface of the component. Alternatively, they are made from metal, thus making a single carbon fiber prototype very expensive due to the mold build. Making the mold using FDM technology can be a smart solution to reduce costs, but due to the layer deposition process, the roughness is quite high. The surface can be improved by reducing the layer height, but it is still not possible to reach the same degree of surface finish of metallic or gelcoat molds without the use of fillers. Thermoplastic polymers, also used in the FDM process, are generally soluble in specific solvents. This aspect can be exploited to perform chemical smoothing of the external surface of a component. The combination of FDM and chemical smoothing can be a solution to produce low-cost molds with a very good surface finish.

## 1. Introduction

The FDM process was first patented by Stratasys in the 1990s to build a three-dimensional plastic object without the use of a mold. The parts are produced layer by layer through the extrusion of thermoplastic filaments usually wound in spools [1]. This is the most popular additive manufacturing technique nowadays as it offers a wide range of thermoplastic material choices from common PLA [2] up to engineering-grade materials such as Nylons [3]. This manufacturing process can be used to create solid components with complex shapes and geometries, as highlighted by the studies of SAVU et al. [4] and Brian et al. [5]. Even though the Additive Manufacturing (AM) processes are challenged because of their low productivity, inferior surface quality, dimensional instability, and the internal anisotropy that decreases the mechanical properties of the products [6]. This process has also shown suitable to produce end-use parts and for small series production [7,8].

### 1.1. 3D Printing for Supporting the Component Manufacturing Process

FDM 3D printing is the most cost-effective additive manufacturing process for thermoplastic materials on the market. This is primarily due to the relative simplicity of hardware construction compared to other technologies. For example, the closed and heated chamber is not always necessary as in the case of Selective Laser Melting (SLM). The filament production is simple, made from polymer granules, and hence there are dozens of manufacturers and a large number of thermoplastic polymers available on the market. The FDM process includes some safety advantages as well, opposite to printing from powder technologies that always require personal protective equipment to avoid problems with the respiratory system during the powder handling and component cleaning. Subsequently, filament 3D printing showed no problems during material handling since the polymer is wound on a spool and is easy to substitute once the filament is finished. Any fumes produced during printing can be effectively removed using specific filters installed on the printer. Nevertheless, the main drawback of FDM printing is related to the surface quality of the component that is lower than that obtained with other 3D printing technologies such as SLM, Stereolithography (SLA) [9], and multi jet fusion from HP [10]. Some research findings suggest that surface noise given by spikes and peaks in the component during modelling could lead to improper print quality [11]. The challenge in correctly predicting residual stresses [12] and deformations of printed components once extruded has so far limited the use of FDM printing for structural components, thus requiring numerous trials before obtaining the finished component with the desired quality level. Overall, FDM printing can be used as a support procedure to create other components, especially in the case of very limited batches or prototypes. Other findings of Komineas et al. [13] can help to accurately calculate overall build time to industrialize elements built with AM. A noteworthy application case is the manufacturing of metal components with the lost wax casting technique [14]. In this case, the starting component on which the ceramic mold is built is no longer made of wax but is 3D printed using low melting thermoplastic polymers such as PLA or ABS [15]. These are suitably modified to reduce the creation of ashes during the polymer removal phase from the mold, which takes place in the furnace. In this case, FDM 3D printing allowed us to avoid the need to create a metal mold (made in aluminum or steel otherwise) in which the wax is casted to obtain the desired model, thus being able to lead to a significant reduction in costs. The alternative to 3D printing would be to make the wax models manually, in which case the printing process allowed us to obtain greater reproducibility and better dimensional tolerances. Additionally, it should be remembered that SLA technology is also used to produce models intended for the traditional lost-wax casting process [16], allowing us in this case to obtain finished components with surface roughness identical to that obtained with wax models. However, the SLA technique is limited to small components due to the large deformations that would occur during the resin printing process and is usually used in the goldsmith and medical sectors [17], and the cost of the resin is also usually higher than that of FDM printing filaments. Another example of how the FDM process can be used as a supporting technology is the mold creation for silicone components [18]. The advantages are similar to the previous example.

### 1.2. Mold to Create Carbon Fiber Components

This study was mainly focused on building a custom mold made through FDM and smoothing processes for polymer-matrix composites. This solution led to a high surface finish that can guarantee the production of continuous fiber-reinforced components such as carbon fiber composites with an epoxy matrix. The mold is necessary, as the current moldless technologies do not allow the production of carbon fiber components starting from a fabric, being constrained to a single, continuous fiber, allowing us just to make reinforcements [19].

Mold manufacturing is usually very expensive. Material options are fiberglass or metal molds. Nevertheless, a starting model is required for the construction of a fiberglass mold; it is usually made by CNC in wood or in high-density polyurethane foam. The model is then covered with a layer of release agent (Polyvinyl Alcohol) or wax. A layer of gelcoat is applied over the model to obtain a shiny and homogeneous surface on the mold, and finally the fibers are soaked in epoxy or a thermosetting resin to give rigidity and consistency to the mold. The whole process has to be done manually by experienced operators and it is difficult to mechanize. The creation of single prototypes in carbon fiber is therefore extremely expensive since it is necessary to make the mold and amortize it with a single piece. The aim of this research is to introduce a solution to this problem using 3D printing technology.

#### FDM 3D Printed Mold

We need to obtain cost-effective, high-quality molds to reduce the costs of prototypes or small batch production. The possibility of making molds with FDM technology is a smart solution [20,21]. The main challenge is related to the high surface roughness that would be transferred directly to the final component. A filler could be used on the mold, followed by manual sandblasting to improve the surface finish. This technique can be applied to components with a relatively simple geometry with low tolerance values, but either way it would still require an important manual intervention.

## 2. Materials and Methods

### 2.1. From Solvent Bonding to Chemical Smoothing

Thermoplastic polymers are generally soluble in some solvent compounds. The application of the solvent on the surface of the plastic component softened its surface. If two components are compressed against each other and the surfaces have been treated with the solvent, a mutual diffusion of the polymer chains is obtained, and the result is a very strong adhesion once evaporated. Solvent bonding differs from adhesive bonding since the solvent does not become permanently adhered to the adhered substrate. A further advantage is that this softening usually occurs well below the glass Transition Temperature (Tg) and therefore the overall component integrity is maintained. This process can also be used to smooth the surface of thermoplastic components [22]. This is superfluous for parts made with injection molding as little surface roughness could be achieved, but instead it could become a process to improve the surface characteristics of a component made by FDM [23]. This process is called chemical smoothing and it allows a localized reaction on the surface of the component only, keeping the main structure unchanged. This process used by research of Kuo et al. [24] can be used to ensure watertight surfaces are achieved on the mold. Once reaching the desired smoothing quality, the component must be cooled in open air or under forced ventilation to promote the evaporation of the solvent from the surface. In this research, the chosen machine for the vapor smoothing is the Polymaker Polishear (Polymaker Inc., Shanghai, China), designed specifically for Polyvinyl butyral (PVB) smoothing using Isopropyl Alcohol (IPA).

### 2.2. Material Choice

**Figure 1:**
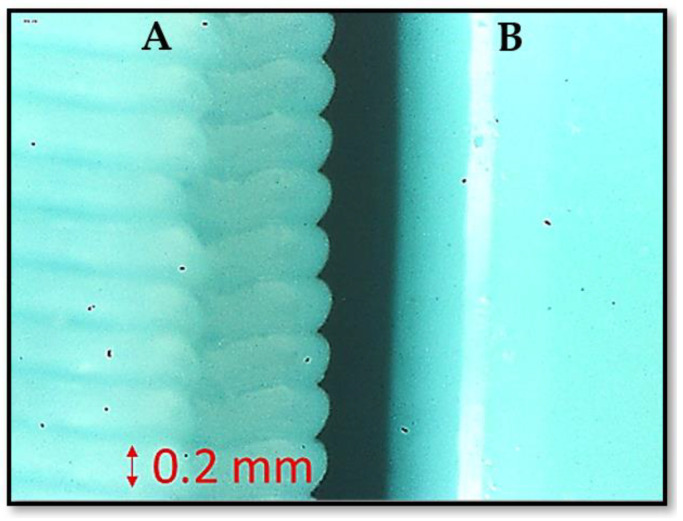
Effect of vapor smoothing, (**A**) the surface as printed, (**B**) same surface after chemical smoothing.

Polyvinyl butyral (PVB) FDM filament (Polymaker Inc., Shanghai, China) is the chosen material for this application. It is the result of a reaction between polyvinyl alcohol and butyraldehyde. This polymer is usually used in the creation of multilayer safety glass [25] in the automotive sector due to its high transparency but is not popular in FDM printing. Currently, there are only two filament manufacturers available, and the cost of this product is higher respect to the most common PLA, but considerably lower than that of Nylon and other engineering materials. Printability is excellent compared to PLA and its mechanical properties are such as the latter [26]. Having a low glass temperature (Tg), the deformations in the printing phase (warping) are limited, even on medium-sized prints, similar to what occurs with PLA. Additionally, similar to every thermoplastic material, it is soluble in a specific solvent, such as IPA alcohol. IPA is a very volatile solvent but it is not very harmful if in contact with human skin. It is sold without special regulations. PLA is also soluble in chloroform [27], but this liquid is much more dangerous and is not for sale. Other polymers such as ABS or ASA could be considered a valid alternative to PVB as they are soluble in acetone [28]. The high Tg of the latter two materials allows the thermal resistance to be greatly increased at the expense of printability. However, ASA and ABS require very high printing and bed temperatures as they require a heated chamber for medium-sized components (150 × 150 × 150 mm). Nevertheless, the onset of warping and delamination phenomena between the layers remains a serious problem. Finally, ASA and ABS contain styrene, which is a toxic component, and the production of fumes during printing could lead to various respiratory system diseases. For safety reasons it is therefore necessary to have a suitable device for filtering the fumes. Overall, PVB offers the best tradeoff between PLA and ABS [29], obtaining the good printability of the former and the solubility of the latter in readily available solvents. In Figure 1 it is possible to see the effect of smoothing in an image taken using an optical microscope at 20× magnification.

### 2.3. Case Study: Manufacturing of a Carbon Fiber Fuel Tap Protection for a Racing Motorbike

The need of light-weight carbon fiber protection is necessary to protect exposed components. This prevents debris or contact with other riders from causing the part to break or malfunction. The fuel cap is particularly exposed in the Husqvarna TC 85 motorbike, and it is therefore necessary to protect it, to avoid dangerous fuel leakage. The need is to produce protection to be installed on that motorbike for the European and World championships. This case study was followed by further components designed to be produced in a very limited series and for the exclusive usage of the team.

#### 2.3.1. Mold Geometry

**Figure 2:**
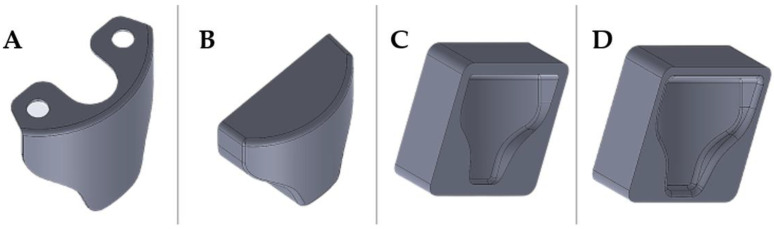
Steps followed during the design of the mold cad model, starting from: (**A**) the actual part, (**B**) making the solid block, (**C**) Cavity extrusion on Mold part, (**D**) Surface-optimized Mold.

The starting point was the CAD drawing of the fuel tap guard. The software used is PTC Creo (PTC Inc., Boston, MA, USA), and the overall dimensions were acquired directly on the fuel tank by means of a caliper. Once the protection geometry was created, a Boolean approach was chosen for the construction of the mold. The CAD file of the protection was modified with the addition of material and draft angles to obtain the correct geometry for the slot on the mold. Finally, the addition of fittings made it possible to avoid ripples in the fabric that could rise to defects in the final component. The overall process is summarized in Figure 2.

#### 2.3.2. Printing Strategy and Settings

**Figure 3:**
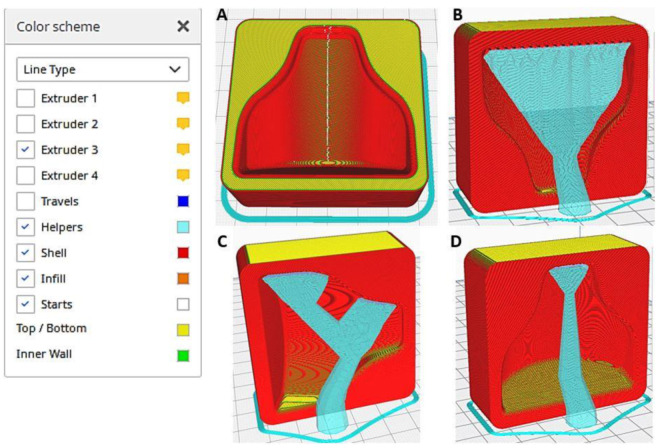
Part orientation on the buildplate, part helpers in blue.

The printing strategy adopted for this component could also be generalized to other parts with similar characteristics. Table 1 shows the printing parameters used to create the component. The first key point is the orientation of the part with respect to the build platform. Although it is a relatively simple component, there are four possible part orientations with respect to the print bed, as shown in Figure 3. The software used for slicing was Cura v4.9.1 (Ultmaker Inc., Zaltbommel, The Netherlands).

Moreover, in the first case (Figure 3A), the mold is placed flat on the printing surface. The idea is to minimize the height of the printed component, thus reducing the total number of layers to be created. This printing mode allowed us to reduce the printing time, but it did not reproduce very well the curvature in the build direction of the component due to the so-called staircase-effect. The action of chemical smoothing can improve the surface roughness of the component and reduce the staircase-effect. In general, it is necessary to obtain the best possible surface prior to treatment in order to reduce the exposure time to solvent vapors. High exposure to the solvent could irreparably damage the surface.

In the second case (Figure 3B), the mold is placed vertically onto the build platform, leading to a time increase of 5% compared to the previous condition, but the staircase-effect problem was significantly improved. As a drawback, many supports were generated, meaning a waste of material and a poor surface finish on supported surfaces. Overall better mold finish quality could be achieved with the use of soluble supports at the expense of a significant price increase for the creation of the mold. Moreover, the result in case 3 (Figure 3C) was similar to the previous case, but the generation of supports was reduced, and the staircase-effect was still present at some points. Finally, the printing position used in the fourth case (Figure 3D) minimized the generation of supports and allowed the maximum resolution of the curvature of the mold.

**Figure 4:**
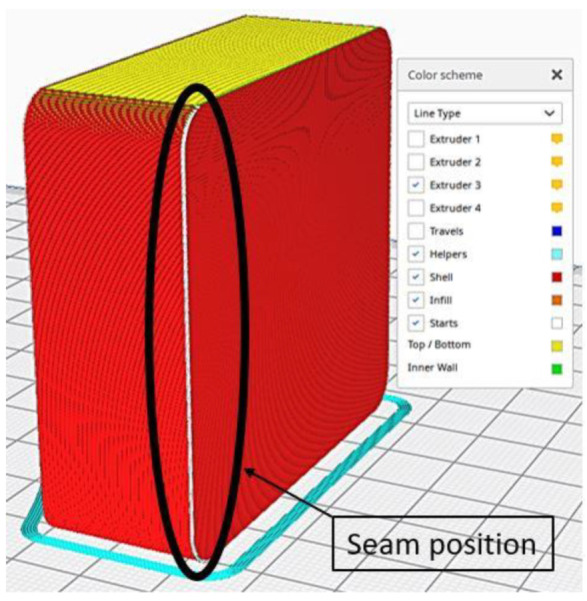
Seam positioning.

**Figure 5:**
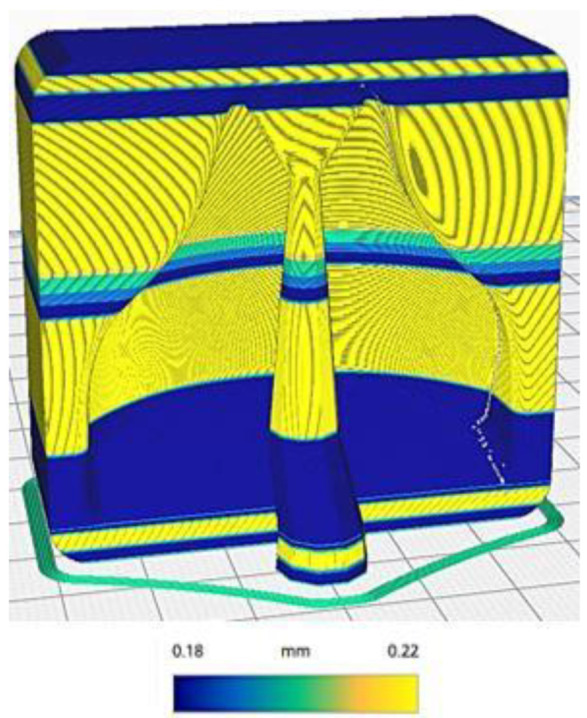
Preview of variable layer height.

However, the settings on Table 1 were adopted to further improve the quality of the mold before the chemical smoothing process to reduce possible imperfections in the cavity. The seam of the outermost wall was preferentially positioned in the rear corner of the mold to avoid seams in the cavity, as seen in Figure 4. Thereafter, it was decided to use a variable layer height, as seen in Figure 5, to further improve the fidelity of the curvature, whilst speed up the printing process at the same time. The molds were not 100% filled; a 20% Gyroid type infill approach with three contour lines were used. This allowed us to obtain molds that could withstand the vacuum lamination process and minimize the material used.

#### 2.3.3. Chemical Smoothing of the Mould Surface

**Figure 6:**
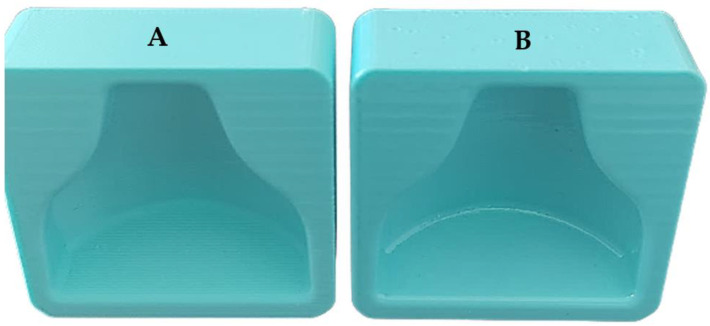
(**A**) Mold immediately after printing; (**B**) mold after smoothing treatment. On the top of the (**B**) mold are small circular spots; these are due to the support surface during smoothing and the rotation of 180 degrees. In the lower part of the mold (**A**) it is possible to see the layers, due to the staircase effect; on the right they have disappeared thanks to the smoothing process.

The mold was initially placed inside the Polyshear device in the same position in which it was printed, and then a 10-min smoothing cycle was performed. Subsequently, it was turned upside down, and a second 10-min smoothing cycle was carried out. Subsequently, two molds were printed with the same printing parameters (thus the same gcode), and both sustained the smoothing process to verify the reproducibility of the process. Both were left to dry for 24 h at ambient temperature. Figure 6 shows the differences before and after the smoothing process.

## 3. Results

### 3.1. Dimensional Verification of a 3D-Printed Mold with an Optical 3D Scanner

**Figure 7:**
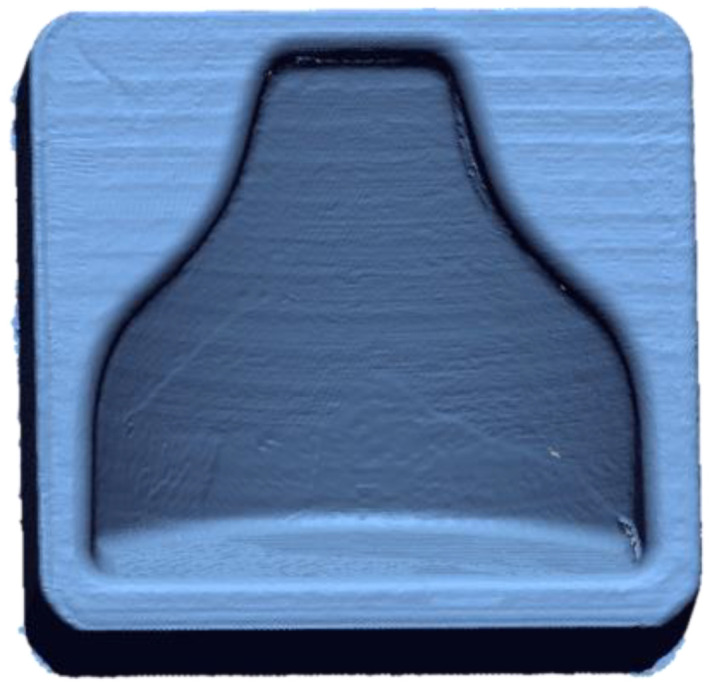
Cloud of point of the first mold.

**Figure 8:**
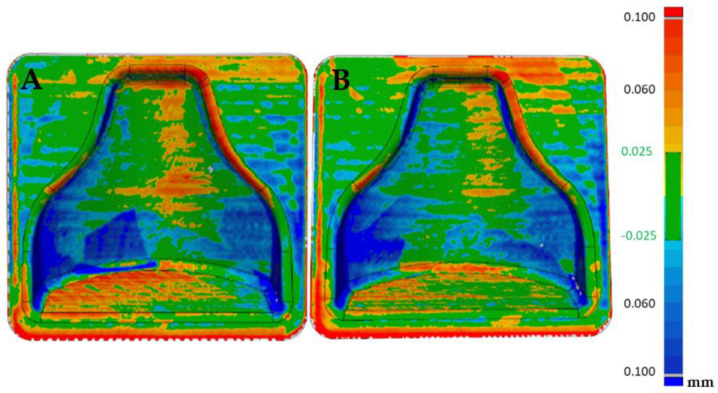
Comparison between the CAD model and first mold (**A**) and CAD model and second mold (**B**).

A Faro 3D scanner was used to check the fidelity of the printed model compared to the designed CAD file. In Figure 7 it is possible to see the cloud of points obtained from the scanning of the first mold. In order to evaluate the reproducibility of this process, two molds with the same gcode were printed. Therefore, a comparison with the theoretical, CAD file could be appreciated in Figure 8, in which the matching was good altogether, as an absolute range of 0.05 mm was obtained in most of the mold. There were areas on the inner boundary highlighted in blue where the matching was not accurate. Nevertheless, the results shown how accurate the printing process was. On the other hand, the overall printing precision could be improved. A so-called loop optimization could be carried out to obtain greater fidelity with the cad file. It could be noticed that although the staircase effect was reduced to a minimum, its effect persisted at some points, where the differences between the cad file and the printed model were higher.

**Figure 9:**
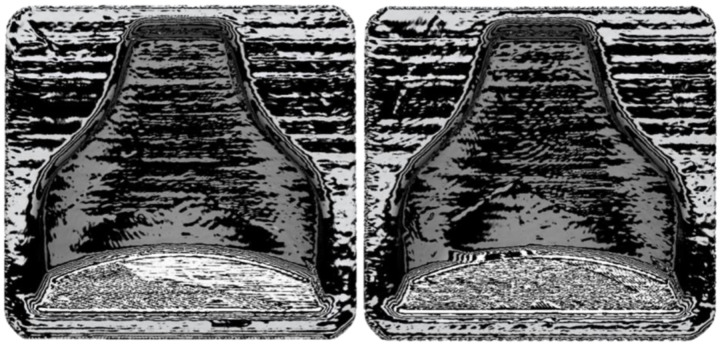
Zebra stripes effect on the two molds after printing, (**left**) mold 1 and (**right**) mold 2.

The visualization tool seen in Figure 9 allowed us to observe small oscillations on the surface of the two molds. These were probably due to the printing parameters and could be improved by reducing acceleration and jerk. These oscillations were less visible in Figure 8 because most result values were in the range of −0.025 +0.025 mm with only the peak values obtained outside this range.

**Figure 10:**
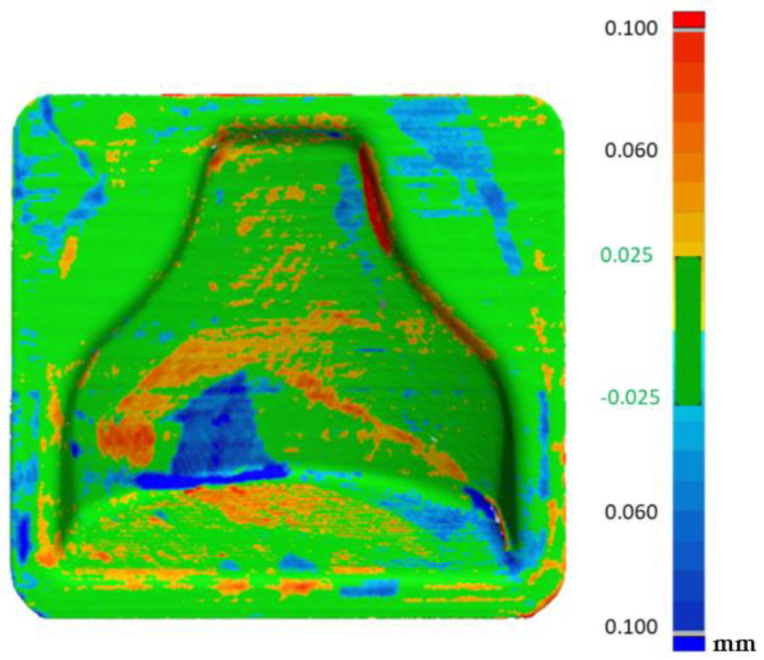
Comparison between the two molds after printing.

Furthermore, Figure 10 shows the comparison between the two molds point-clouds with each other. It could be seen that the reproducibility of printing with this specific material was very high. In fact, the two molds could be superimposed, as can be seen from the almost complete green color of the image.

### 3.2. Dimensional Verification of the 3D-Printed, Chemical-Smoothed Mold with an Optical 3D Scanner

**Figure 11:**
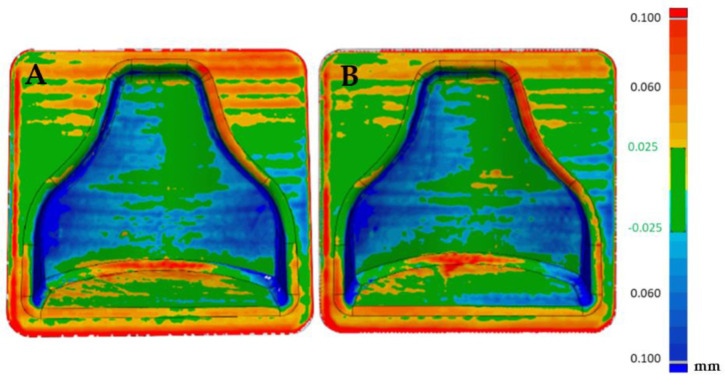
Comparison between the CAD model and first mold after chemical smoothing (**A**) and cad model and second mold after chemical smoothing (**B**).

After the first dimensional verification, both molds were subjected to a chemical smoothing process, as described in Section 2.3.3. Afterwards, dimensional verification with respect to the CAD file gave the results visible in Figure 11. As expected, the chemical smoothing process reduced the peaks of innacuracies and filled the valleys, reducing the overall external dimensions of the component. Once again, the verification was carried out on both molds printed to evaluate the repeatability of the process, and a comparison was made with respect to the CAD file values and with respect to the scanning performed before the smoothing process.

**Figure 12:**
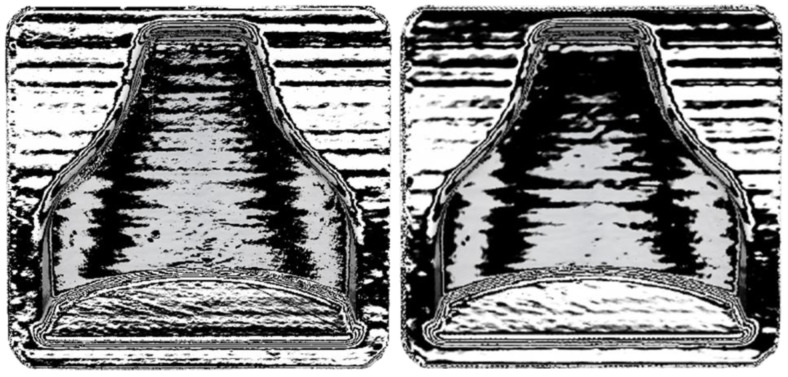
Zebra stripes effect on the two molds after printing. (**left**) mold 1 and (**right**) mold 2.

**Figure 13:**
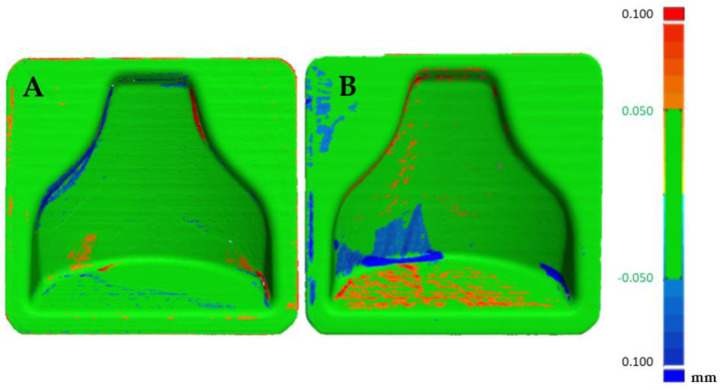
Comparison between the scan of mold 1 pre and post smoothing (**A**) and comparison of mold 2 pre and post smoothing (**B**).

**Figure 14:**
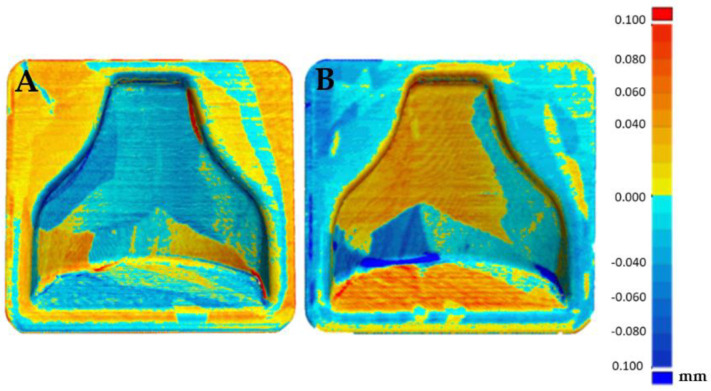
Comparison between the scan of mold 1 pre and post smoothing (**A**) and comparison of mold 2 pre and post smoothing (**B**).

Afterwards, thanks to the Geomagic visualization tool (3d systems Inc., Valencia, CA, USA), a comparison with Figure 9 was performed in Figure 12, and it was possible to see a greater homogenization of the surfaces, which can be seen from the zebra stripes. This was due to the smoothing process that turned the surfaces smoother and glossier. Additionally, it was possible to appreciate the effect of smoothing on the dimensional variation of the component in Figure 13 and Figure 14. The molds before and after treatment were compared.

From Figure 13 it could be deduced that overall, the dimensional tolerance of the component after smoothing reached an absolute value of 0.1 mm, with both molds completely colored green. Figure 14 shows that the treatment was uniform; positive or negative values also differed in the way in which the software superimposed the two scans and did not indicate removal or addition of material. Therefore, they must be understood in an absolute manner. In fact, the software tried to minimize the distance between the points of one mesh and those of the other, obtaining the result shown.

**Figure 15:**
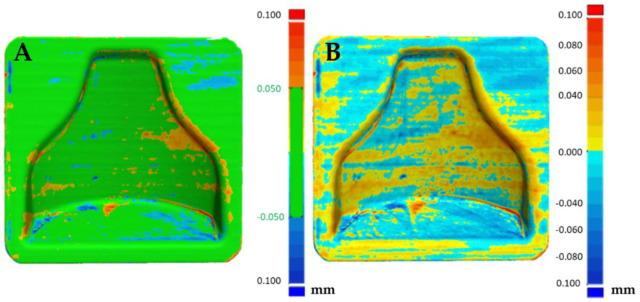
Comparison between the two molds (mold 1 (**A**), mold 2 (**B**)) after smoothing.

In Figure 15A the reproducibility of the process can be evaluated. In fact, it can be seen that the two post-treatment molds differed in an absolute value by less than 0.1 mm, with the likely analysis almost completely colored green. Figure 15B shows the areas that were slightly positive and those that were negative, but overall a good matching was obtained, making it possible to guarantee narrow tolerances.

### 3.3. Carbon Fiber Vacuum Lamination Process

**Figure 16:**
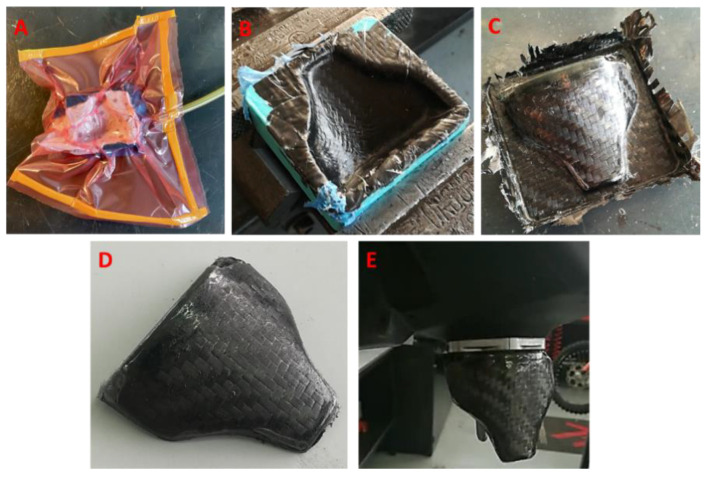
Carbon fiber component production steps: (**A**) component lamination and vacuum, (**B**) mold opened after resin curing, (**C**) component just extracted from the mold, (**D**) trimmed and finished component, (**E**) component mounted on the tank.

The mold in this study was used to create a part with the vacuum lamination process to make the component of Figure 2A. It is worth noticing that the FDM-produced mold did not require the use of a release agent, unlike conventional fiberglass and gelcoat or metal molds. Therefore, a resin-impregnated carbon fiber cloth was placed directly in the cavity of the mold, after which a layer of peel ply fabric and a layer of absorbent tissue were placed and the vacuum was made. The resin used was AERO68^®^ (Rius Composites SRL, Italy)), suitable for wet-layup laminations at room temperature with carbon fiber, glass fiber, and Kevlar. The component was then extracted from the mold with the aid of a plastic wedge and then finished and mounted on the fuel tank. Figure 16 shows a series of images that summarize the production process of the component.

## 4. Conclusions

The procedure highlighted proved to be valid for creating carbon fiber components produced in very small numbers. The choice of using molds printed in FDM allowed us to reduce time and costs considerably compared to conventional methods. The need to obtain a smooth surface of the final component is also fundamental to guarantee optical and mechanical properties. The chemical smoothing allowed us to obtain a smooth surface, without using gelcoat or other products/techniques for reducing surface roughness. Overall, the quality of the final component was high, but there are margins for improvement, especially in terms of dimensional tolerances. By knowing the effect of the smoothing process at a dimensional level approximately, it is possible to modify the starting cad file to obtain more accurate final dimensions of the mold.

The printing parameters used for the creation of the mold could also be improved. The correct setting of printing parameters aimed at obtaining a better surface quality, therefore guaranteeing shorter smoothing times, and reaching higher dimensional tolerances. These ensure less alcohol absorption by the surface and therefore less time to get the component finished and ready to be used.

## 5. Future Developments

Future developments include further printing methodologies for this material, i.e., the adoption of a lower layer height, that would lead to much longer printing times but an even higher surface quality.

Additional tests of this element are needed, e.g., if it would be possible to make the entire mold in cheap PLA and only the outer layer in PVB, after verifying a good adhesion between the two materials. A possible alternative to PLA could be PETG, in order to further reduce the costs of a single mold and take full advantage of the existing technologies of 3D FDM printing and the smoothing technique.

Further research is needed for the vapor-process parameters and how to influence the surface roughness and the actual fabrications of parts with other materials. Mechanical tests could be carried out to evaluate the mechanical properties of the carbon fiber components obtained with this technology.

## Figures and Tables

**Table 1:** Applied Slicing Printing Parameters in Cura.

Parameter	Value	Unit
Layer Height	0.22	mm
Line width	0.4	mm
Wall Line Count	3	-
Z seam position	Back Left	-
Top Layers	3	-
Bottom Layers	3	-
Infill Density	15	%
Infill Patten	Gyroid	-
Printing Temperature	205	°C
Build Plate Temperature	65	°C
Flow	100	%
Print Speed	80	mm/s
Travel Speed	250	mm/s
Retraction Distance	4	mm
Fan Speed	70	%
Regular Fan Speed at Height	0.2	mm
Support Structure	Tree	-
Support Overhang Angle	60	°
Adaptative Layers Maximum Variation	0.02	mm
